# Azithromycin Failure in *Mycoplasma genitalium* Urethritis

**DOI:** 10.3201/eid1207.051558

**Published:** 2006-07

**Authors:** Catriona S. Bradshaw, Jorgen S. Jensen, Sepehr N. Tabrizi, Timothy R.H. Read, Suzanne M. Garland, Carol A. Hopkins, Lorna M. Moss, Christopher K. Fairley

**Affiliations:** * Alfred Hospital, Victoria, Australia;; † University of Melbourne, Victoria, Australia;; ‡ Statens Serum Institut, Copenhagen, Denmark;; § Royal Women's Hospital, Victoria, Australia

**Keywords:** non-gonococcal urethritis, Mycoplasma genitalium, azithromycin, moxifloxacin, dispatch

## Abstract

We report significant failure rates (28%, 95% confidence interval 15%–45%) after administering 1 g azithromycin to men with *Mycoplasma genitalium*–positive nongonococcal urethritis. In vitro evidence supported reduced susceptibility of *M. genitalium* to macrolides. Moxifloxacin administration resulted in rapid symptom resolution and eradication of infection in all cases. These findings have implications for management of urethritis.

*Mycoplasma genitalium* has been well described as a pathogen in men with acute and chronic nongonococcal urethritis (NGU) and has been associated with cervicitis in women (*1*). Since culturing the organism is difficult, limited information has been available regarding its antimicrobial drug susceptibility. In vitro studies suggest it is susceptible to tetracyclines, macrolides, and fluoroquinolones (*2**–**4*), although reduced susceptibility to tetracyclines (*5*) and specific fluoroquinolones has been reported (*4**,**6*). In clinical studies, doxycycline and levofloxacin (*4**,**7**–**11*) have substantial failure rates, whereas early reports suggest single-dose azithromycin may be more efficacious (*10**,**11*). Treatment guidelines for acute NGU include 1 g single dose of azithromycin or doxycycline for 7 days, but no evidence-based guidelines exist for treatment of *M. genitalium*–positive NGU.

We report treatment failure of single-dose and multidose azithromycin therapy in *M. genitalium*–positive NGU and provide in vitro evidence of macrolide resistance in clinical isolates. Persistent infection was eradicated with moxifloxacin.

## The Study

Cases were derived from a case-control study of acute NGU conducted from March 2004 to March 2005 at Melbourne Sexual Health Centre (MSHC), Australia (*12*). Participants completed a questionnaire, underwent examination, and had first-void urine samples analyzed by strand-displacement amplification (ProbeTec-ET CT-Amplified-DNA-Assay, Becton, Dickinson and Company, Sparks, MD, USA) for *Chlamydia trachomatis* and by polymerase chain reaction (PCR) for *M. genitalium* (*13*), herpes simplex viruses (HSV-1 and -2), *Trichomonas vaginalis*, *Ureaplasma urealyticum* and *parvum*, *Gardnerella vaginalis*, and adenoviruses (*12*). Culture of urethral samples in modified-Thayer-Martin medium was performed for *Neisseria gonorrhoeae*.

Men with *M. genitalium* infection were instructed regarding partner notification and reinfection and were asked to return for a test of cure (TOC) 1 month posttreatment. Men with persistent *M. genitalium* infection were given 1 g single dose of azithromycin or 1 g weekly for 3 doses, but after apparent failure of azithromycin therapy in 3 men without reinfection, participants with persistent infection were offered moxifloxacin, 400 mg daily for 10 days. Four urethral specimens from men for whom azithromycin therapy failed were inoculated into SP4 medium, frozen (–80°C), and shipped on dry ice to Statens Serum Institut, Denmark, for culture in Vero cells and antimicrobial drug susceptibility testing (*6*). *M. genitalium* strains in Vero cell culture were grown in the presence of different concentrations of antimicrobial drugs, and growth of *M. genitalium* was monitored by quantitative PCR for determination of MIC (*6*).

The Human Research and Ethics Committee of the Alfred Hospital, Victoria, approved the study. Data were stored in Microsoft Access and analyzed by using SPSS version 12 (SPSS Inc., Chicago, IL, USA). Ninety-five percent confidence intervals (CIs) were calculated for proportions, which were compared by using the Fisher exact test. Patients were excluded from the analysis when information or specimens were not available.

*M. genitalium* was detected in 31(9.4%) of 329 patients (95% CI 6.6%–12.9%) and 3 of 307 controls. No patients with *M. genitalium* infection had other pathogens detected (*12*). Men with *M. genitalium* had a median age of 33 years (range 22–54 years); 25 were heterosexual, and 9 were homosexual (behavioral and clinical data are presented elsewhere [12]). Six female and 4 male asymptomatic sexual contacts of *M. genitalium*–infected men were tested; a throat and anal sample in 1 man and a cervical sample in 1 woman were positive for *M. genitalium*. Contacts were presumptively treated with 1 g single dose of azithromycin; however, the infected male contact required moxifloxacin after azithromycin treatment failed in this patient and in the index patient.

Thirty-two men (94%) completed their TOC, a median of 31 days (range 17–59 days) after receiving azithromycin. Twenty-three (72%) men had a negative TOC (95% CI 55%–85%) and were asymptomatic; however, 9 (28%, 95% CI 15%–45%) were positive for *M. genitalium* by PCR. No treatment failures reported unprotected sexual contact posttreatment or previous antimicrobial drugs. Azithromycin treatment failed in 4 (44%, 95% CI 16%–76%) homosexual males, compared to 5 (22%, 95% CI 8%–42%) heterosexual males (p = 0.23). Five men for whom azithromycin treatment failed reported sexual contact with partners from Asia before symptom onset (56%, 95% CI 24%–84%) compared to 6 azithromycin responders (27%, 12%–48%), p = 0.22. Eight patients for whom azithromycin treatment failed reported an initial reduction or resolution of symptoms following azithromycin and then experienced recurrent urethral symptoms; 1 male was persistently asymptomatic. The Table outlines urethral Gram stain findings and treatment of men with persistent infection; all 8 men became asymptomatic after receiving moxifloxacin.

**Table:** Case-patients experiencing single-dose azithromycin treatment failure*

Patient†		TOC-1			TOC-2			TOC-3			TOC-4
	Pretreatment PMN/HPF	PMN/HPF	Mg PCR	Treatment-2	PMN/HPF	Mg PCR	Treatment-3	PMN/HPF	Mg PCR	Treatment-4	Mg PCR
1‡	>5	>5	Pos	1 g AZI	>5	Pos	1 g AZI weekly, 3 doses	<5	Pos	MOX, 400 mg daily 10 d	Neg
2‡	>5	<5	Pos	1 g AZI	§	Pos	1 g AZI weekly, 3 doses	§	Pos	Moxifloxacin, 400 mg daily 10 d	Neg
3	<5	§	Pos	1 g AZI weekly 3 doses	<5	Pos	MOX, 400 mg daily 10 d	§	Neg		
4‡¶	>5	>5	Pos	MOX, 400 mg daily 10 d		Neg					
5‡	>5	>5	Pos	MOX, 400 mg daily 10 d		Neg					
6	<5	§	Pos	MOX, 400 mg daily 10 d		Neg					
7#	>5	<5	Pos	MOX, 400 mg daily 10 d		Neg					
8	>5	<5	Pos	MOX, 400 mg daily 10 d		Neg					
9	>5	>5	Pos	MOX, 400 mg daily 10 d		Neg					

*PMN/HPF, polymorphonuclear count per high power field (×1,000 magnification); Mg, *Mycoplasma genitalium*; PCR, polymerase chain reaction; Pos, positive; Neg, negative; AZI, azithromycin; MOX, moxifloxacin; d, days; bd, twice daily; tests of cure (TOCs) were performed 1 month after commencement of each therapy; TOC-1, first test of cure 1 month after treatment. †All men treated with 1 g of azithromycin at first examination. ‡Patients with specimens cultured, MIC data available and presented for all 4 isolates. §Urethral PMN count not available. ¶Patient 4 saw his general practitioner 3 weeks after receiving 1 g azithromycin with recurrent urethral discharge and dysuria and was retreated with 1 g azithromycin before his TOC-1. #Only patient who was asymptomatic with persistent infection.

The 4 TOC specimens from men with azithromycin failure available for culture yielded growth of *M. genitalium*. Antimicrobial drug susceptibility testing showed increased MICs to macrolides: azithromycin >8 mg/L, erythromycin >32 mg/L, and clarithromycin >32 mg/L. All isolates were susceptible to moxifloxacin (MIC range 0.031–0.125 mg/L) and could be considered susceptible to doxycycline (MIC range 0.125–0.25 mg/L). However, correlates between in vitro MICs and treatment efficacy have not yet been established.

## Conclusions

The azithromycin failure rate in *M. genitalium*–positive NGU was 28% (15%–45%) in this study and was associated with recurrent urethral symptoms in 8 of 9 cases. Longer course azithromycin ameliorated but did not resolve symptoms or eradicate infection, whereas moxifloxacin resulted in rapid symptom resolution and eradicated infection. Symptom improvement followed by recrudescence has been reported after levofloxacin failure (*9*). Culture of *M. genitalium* from all 4 specimens, and reduced susceptibility to azithromycin in vitro, demonstrates that azithromycin-resistance rather than reinfection caused treatment failure and that nonviable DNA was not the reason for a persistently positive PCR. The availability of strains in pure culture will enable investigation into resistance mechanisms, and work in progress indicates that mutations in region-V of the 23S-rDNA explain the azithromycin resistance (J.S. Jensen, unpub. data).

*M. genitalium* has been associated with persistent NGU (*1*). Recent data indicate that sequence variation in the gene mediating adhesion to epithelial cells coincides with the immune response in patients and that changes in this gene occur rapidly with persistent infection (*14*). In vitro studies also suggest that macrolide-resistant mutants can be selected by serial passage of mycoplasmas in subinhibitory concentrations of macrolide (*15*). Macrolide resistance in our study could have been induced by single-dose azithromycin, which may be suboptimal for eradication of a slow-growing bacterium such as *M. genitalium*. Studies are ongoing to establish whether resistance in our isolates was present pretreatment or emerged after azithromycin-exposure. It is possible that initial use of higher doses or longer durations of azithromycin in *M. genitalium*-positive NGU could avoid selection of resistant mutants. The association between azithromycin failure and sexual partners from Asia may be clinically relevant, given the high levels of antimicrobial drug resistance reported in other sexually transmitted infections such as *Neisseria gonorrhoeae* infections in Asia, and the higher failure rates seen in homosexual men, while not statistically significant, may represent a core-group effect.

Azithromycin or doxycycline is recommended treatment for NGU. While treatment-failure in *M. genitalium*–positive NGU appears common with doxycycline (*4**,**7**–**11*), early reports suggest 1 g azithromycin is more effective, with cure rates of 85% (*10**,**11*), and that prolonged azithromycin treatment (500 mg on day 1 and 250 mg on days 2–5) eradicates *M. genitalium* in 95% of cases (*10*). However, if treatment-failure after 1 g azithromycin is as prevalent as indicated by our study in *M. genitalium*–positive NGU, this has implications for the use of single-dose azithromycin as first-line treatment for NGU and leaves few evidence-based treatment options. Information regarding sensitivity of *M. genitalium* to fluoroquinolones has been limited, but reports suggest differential activity against *M. genitalium*, with levofloxacin (*4**,**9*) less active than gatifloxacin, sparfloxacin, and tosufloxacin in vitro and in vivo and moxifloxacin more active than levofloxacin and ciprofloxacin in vitro (*6*).

We report significant failure rates of azithromycin in *M. genitalium*–positive NGU that is supported by in vitro evidence of reduced susceptibility to macrolides. Recurrent urethral symptoms following azithromycin therapy only occurred in persons with persistent *M. genitalium* infection and resolved with moxifloxacin.

Because single-dose azithromycin is recommended treatment for NGU, these findings have implications for treatment guidelines and highlight the need for randomized studies to determine optimal treatment for *M. genitalium*–positive NGU and *M. genitalium* infection in women, who are at high risk for sequelae.

## References

[1] Jensen JS. *Mycoplasma genitalium*: the aetiological agent of urethritis and other sexually transmitted diseases. J Eur Acad Dermatol Venereol. 2004;18:1–11. 10.1111/j.1468-3083.2004.00923.x 14678525

[2] Hannan PC, Woodnutt G. In vitro activity of gemifloxacin (SB 265805; LB20304a) against human mycoplasmas. J Antimicrob Chemother. 2000;45:367–9. 10.1093/jac/45.3.367 10702559

[3] Duffy LB, Crabb D, Searcey K, Kempf MC. Comparative potency of gemifloxacin, new quinolones, macrolides, tetracycline and clindamycin against *Mycoplasma* spp. J Antimicrob Chemother. 2000;45(Suppl 1):29–33. 10.1093/jac/45.suppl_3.29 10824029

[4] Yasuda M, Maeda S, Deguchi T. In vitro activity of fluoroquinolones against *Mycoplasma genitalium* and their bacteriological efficacy for treatment of *M. genitalium*-positive nongonococcal urethritis in men. Clin Infect Dis. 2005;41:1357–9. 10.1086/496983 16206116

[5] Hannan PC. Comparative susceptibilities of various AIDS-associated and human urogenital tract mycoplasmas and strains of *Mycoplasma pneumoniae* to 10 classes of antimicrobial agent in vitro. J Med Microbiol. 1998;47:1115–22. 10.1099/00222615-47-12-1115 9856648

[6] Hamasuna R, Osada Y, Jensen JS. Antibiotic susceptibility testing of *Mycoplasma genitalium* by TaqMan 5´ nuclease real-time PCR. Antimicrob Agents Chemother. 2005;49:4993–8. 10.1128/AAC.49.12.4993-4998.2005 16304163 PMC1315946

[7] Falk L, Fredlund H, Jensen JS. Tetracycline treatment does not eradicate *Mycoplasma genitalium.* Sex Transm Infect. 2003;79:318–9. 10.1136/sti.79.4.318 12902584 PMC1744713

[8] Johannisson G, Enstrom Y, Lowhagen GB, Nagy V, Ryberg K, Seeberg S, Occurrence and treatment of *Mycoplasma genitalium* in patients visiting STD clinics in Sweden. Int J STD AIDS. 2000;11:324–6. 10.1258/0956462001915958 10824941

[9] Maeda SI, Tamaki M, Kojima K, Yoshida T, Ishiko H, Yasuda M, Association of *Mycoplasma genitalium* persistence in the urethra with recurrence of nongonococcal urethritis. Sex Transm Dis. 2001;28:472–6. 10.1097/00007435-200108000-00010 11473221

[10] Bjornelius E, Anagrius C, Bojs G, Hans G, Johanisson G, Lidbrink P, *Mycoplasma genitalium*: when to test and treat. Present status in Scandinavia. In: 15th Biennial Meeting of the International Society for Sexually Transmitted Diseases Research. Ottawa, Canada; 2003 [cited 2006 May 22]. Available from http://www.isstdr.org

[11] Mroczowski TF, Mena LA, Nsumai M, Martin DH. A randomized comparison of azithromycin and doxycycline for the treatment of *Mycoplasma genitalium*-positive urethritis in men. In: 16th Biennial Meeting of the International Society for Sexually Transmitted Diseases Research. Amsterdam, the Netherlands; 2005 [cited 2006 May 22]. Available from http://www.isstdr.org 10.1086/599033 19438399

[12] Bradshaw CS, Tabrizi SN, Read TRH, Garland SM, Hopkins CA, Moss LM, Etiologies of non-gonococcal urethritis: bacteria, viruses and the association with oro-genital exposure. J Infect Dis. 2006;193:336–45. 10.1086/499434 16388480

[13] Yoshida T, Deguchi T, Ito M, Maeda S, Tamaki M, Ishiko H. Quantitative detection of *Mycoplasma genitalium* from first-pass urine of men with urethritis and asymptomatic men by real-time PCR. J Clin Microbiol. 2002;40:1451–5. 10.1128/JCM.40.4.1451-1455.2002 11923372 PMC140368

[14] Iverson Cabral SL, Astete SG, Cohen CR, Totten PA. Heterogeneity of the MGPB gene in *Mycoplasma genitalium*, a mechanism for persistence. In: 16th Biennial meeting of the International Society for Sexually Transmitted Diseases Research, Amsterdam, the Netherlands; 2005 [cited 2006 May 22]. Available from http://www.isstdr.org

[15] Pereyre S, Guyot C, Renaudin H, Charron A, Bebear C, Bebear CM. In vitro selection and characterization of resistance to macrolides and related antibiotics in *Mycoplasma pneumoniae.* Antimicrob Agents Chemother. 2004;48:460–5. 10.1128/AAC.48.2.460-465.2004 14742195 PMC321523

